# Osteo-myelitis of the Tibia and Fibula

**Published:** 1896-10

**Authors:** E. B. Osborn

**Affiliations:** Cleburne, Texas


For the Texas Medical Journal.

### OSTEO-MYELITIS OF TIBIA AND FIBULA.

BY E. B. OSBORN, M. D., CLEBURNE, TEXAS.

Read before the Johnson County Medical Association, Thursday, September 3rd, 1896.

THE specimen I present for your examination to-night is the distal end of the right leg of L. H., a lad nine years of age, the amputation being done for osteo-myelitis of tibia and fibula, with subsequent periostitis of both bones, and anchylosis of the knee joint. Especially would I call your attention to the amount of necrosis that has taken place here, and the great number of fistulæ leading from the diseased bone.

Osteo myelitis is a term which is best limited to diffuse inflammation of the lining membrane and the medulla of a bone with implication of the surrounding cancellous tissue. Such inflammation is always of septic origin, and is closely allied to cellulitis of the soft parts, being caused by, or associated with, in the majority of cases, the tubercle bacillus.

However, it is not absolutely essential that the bacillus of tuberculosis should be found in these cases, as Dr. Becker has recently demonstrated in the laboratory of the Berlin Imperial Sanitary office, that the micro-organisms discovered by Schuller and Rosenbach in these cases of acute osteo-myelitis, when properly cultivated, would produce the identical disease when inoculated in animals, *provided* the bone had been previously fractured or injured so as to furnish a suitable media for the organism when it reached the part through the blood stream—the organism having been injected in the jugular vein.

Further study of this micrococcus shows it to be identical with micrococcus pyogenes aurens.

Osteo-myelitis is characterized by the rapid formation of pus which infiltrates the neighboring cancellous tissue, and is apt to extend along the whole shaft, not only in a longitudinal direction, but, also, through a varying thickness of the surrounding osseous structure, in many cases reaching the surface and affecting the periosteum. Of the septic inflammations of bone, two chief varieties present themselves:

*First.* The one associated with an *open wound*, originating, ordinarily, in the shaft of a long bone and generally met with in adults.

*Second.* One without such an associated wound, beginning in the part near the epiphyseal line, arising, as I have said, not through an open wound, or through traumatism, but by the pyogenic organism having been brought to the affected area by the blood stream.

This latter variety is said to be peculiarly a disease of childhood, the effect produced being variable. In a comparatively small proportion of the cases intense and rapidly destructive inflammation of the bone follows. It is stated that this disease has its starting point in the tissues near an epiphyseal line, due to the frequency of juxta-epiphyseal strains, the femur and fibia being the bones most often affected.

On July 9th, 1895, I was consulted by the father of the lad, he stating that for several days his son had complained of pain just about one inch below the knee joint; that he had suffered from a chill, and was now having fever, the pain having started just after the chill.

Prior to this time his son had been healthy, suffering none at all; playing day after day with the children of the neighborhood, but since the chill he had never been able to use the limb at all.

Inquiry into the patient's family history showed it to be entirely free from any tubercular or scrofulous disease; coming then to the patient's history, I found he had suffered no traumatism, and the disabled member then complained of was not at that time visably affected, however, I refused to prescribe, and requested that the patient be brought to my office.

On examining him three days later, I found the parts about the leg inflamed and odematous, sensitive to pressure, and a complete inability, on the part of the patient, to move the limb by its own muscles, and the superficial veins abnormally distended, fluctuation was present, and to my mind indicated pus, consequently I put the patient on the table and made a deep, longitudinal incision, drawing off about one and one-half pints of pus, which was very thick and odorous; on probing, I detected the presence of dead bone, and the periosteum was lifted off the shaft for a variable distance.

After evacuating all the pus and irrigating with hot sterile water, followed by bichloride of mercury irrigations, 1 to 1000, the wound was packed with sterilized iodoform gauze, and the little fellow put to bed, instructing them to feed him the most nourishing foods, giving also cod liver oil and good whisky.

From that time on he has complained of no pain at all, and has had no opiate. Three days later I found the leg swelling lower down, so I made several counter openings along the shaft of the bone, irrigated thoroughly with bichloride of mercury, 1 to 1000, then peroxide of hydrogen, full strength, and packed with 10 per cent. iodoform gauze.

This procedure was kept up daily, and fresh dressings applied each time, yet we continued to get quantities of purulent pus. Finding that these methods had no effect in allaying the inflammation, we irrigated with permanganate of potash solutions, 1 to 500, and painted the external parts with tincture of iodine, but as this did not check the trouble, and pieces of bone were daily passing out through the fistullæ and openings, we again irrigated with bichloride solution and removed as much dead bone as was perceptible; very soon, however, the knee joint commenced to swell, and rapidly grew œdematous, anchylosis soon taking place, hence I advised amputation, but the family objected, and insisted that I should continue the former plan of treatment, so on August 18, 1895, with my patient fully anesthetised, with the assistance of my father, Dr. J. D. Osborn, I laid the knee joint open, both externally and internally, gnawing away all dead tissues, and drained the joint through both openings, and irrigated with the solutions before mentioned.

The patient seemed to improve after this, and drifted along until September, when my duties called me away from this city, but I saw the patient again in January, 1896, and found the pus coming away in great quantities, notwithstanding, the dressings, draining and irrigations had been continually kept up, and the case treated under the strictest antiseptic rules.

When I returned to Cleburne in May the case again came under my observation, and on examination I found a small fistula about one inch above the knee joint, which the probe showed to lead to the femur, and small pieces of bone were from time to time passing away through it.

At this point I stood pat for amputation, although the inguinal glands of that side showed decided involvement, the patient weak and anæmic.

Consequently on July 7, 1896, with the assistance and in the presence of Drs. Greenwell, Strickland, Gregory and J. D. Osborn, after thoroughly carrying out our antiseptic technique, I amputated at the middle third of the thigh, doing a modified circular amputation, as that operation afforded the best flaps under the circumstances, and I was enabled to save more stump than by any other possible operation.

The patient rallied nicely from the shock, and has gained flesh daily, has no pain whatever, nor has he had since the operation, as we had no sepsis, taking every possible precaution, even to using silk-worm gut as sutures.

There are two things, gentlemen, connected with this case that I regret. First. I should have examined this pus microscopically for the tubercle bacillus, which would have been very little trouble as I am constantly making examinations of this character.

Second, *I should have amputated earlier* and saved more of the limb, as the instrument makers are constantly admonishing the profession to save as much of the limb as possible, and I assure you that I shall advise early amputations in those cases of osteo-myelitis coming under my observation, and refusing to quickly succumb to the stereotyped antiseptic treatment, combined with removing the necrossed bone and drainage.

